# *In vivo* dosimetry in pelvic brachytherapy

**DOI:** 10.1259/bjr.20220046

**Published:** 2022-08-10

**Authors:** Orla Anne Houlihan, Geraldine Workman, Alan R Hounsell, Kevin M Prise, Suneil Jain

**Affiliations:** Department of Clinical Oncology, Northern Ireland Cancer Centre, Belfast Health and Social Care Trust, Belfast, UK; Patrick G. Johnston Centre for Cancer Research, Queen's University Belfast, Belfast, UK; Radiotherapy Physics, Northern Ireland Cancer Centre, Belfast Health and Social Care Trust, Belfast, UK; Patrick G. Johnston Centre for Cancer Research, Queen's University Belfast, Belfast, UK; Radiotherapy Physics, Northern Ireland Cancer Centre, Belfast Health and Social Care Trust, Belfast, UK; Patrick G. Johnston Centre for Cancer Research, Queen's University Belfast, Belfast, UK; Department of Clinical Oncology, Northern Ireland Cancer Centre, Belfast Health and Social Care Trust, Belfast, UK; Patrick G. Johnston Centre for Cancer Research, Queen's University Belfast, Belfast, UK

## Abstract

Brachytherapy is an effective treatment in the curative management of prostate and gynaecological cancers. With advances in technology, brachytherapy has increased in complexity in recent years. Human error, equipment malfunction, patient organ motion and radioactive source displacement can result in substantial deviation of delivered dose from planned dose. To limit adverse clinical outcomes, adequate steps to improve the robustness of pathway processes, ensure the implementation of appropriate treatment margins and confirm the delivered dose must be considered. *In vivo* dosimetry is one such method of dose validation which, if implemented appropriately within clinical practice, is an attractive technique for reducing dosimetric uncertainties and identifying potential errors. This review aims to describe the dosimetric uncertainties and potential errors associated with brachytherapy, the potential for *in vivo* dosimetry in adaptive brachytherapy as a key method of dose validation, and the clinical considerations and future directions of *in vivo* dosimetry.

**Advances in knowledge:**

This paper describes the potential role for *in vivo* dosimetry in the reduction of uncertainties in pelvic brachytherapy, the pertinent factors for consideration in clinical practice, and the future potential for *in vivo* dosimetry in the personalisation of brachytherapy.

## Introduction

The use of ionising radiation for radiotherapy is an effective cancer treatment strategy which induces cancer cell death through direct and indirect DNA damage.^1^ In brachytherapy, radioactive sources such as strontium-90 (^90^Sr), iridium-192 (^192^Ir) and iodine-125 (^125^I) which emit radiation in the form of β particles (^90^Sr)^2^ or γ rays (^192^Ir, ^125^I)^3^ are directly inserted within or in close proximity to the radiotherapy target. These are permanently inserted in the case of low dose rate (LDR) brachytherapy sources (*e.g.* ^125^I) which emit radiation at <2 Gy per hour or inserted for a short period of time in the case of high dose rate (HDR) brachytherapy sources (*e.g.* ^192^Ir) which emit radiation at >12 Gy per hour.^4,5^

Brachytherapy is a common method of treatment for prostate and gynaecological malignancies and has several advantages over external beam radiotherapy (EBRT). These include the ability to deliver a much higher dose of radiation directly to the cancer.^4^ Internal source placement with brachytherapy is associated with rapid radiation dose fall-off as a result of the inverse square law. This advantageous dose distribution improves the therapeutic ratio resulting in the capability of delivering higher radiation doses to the tumour and/or reduced dose to adjacent organs at risk (OARs) compared with EBRT, thereby increasing the probability of cure and/or reducing the likelihood of adverse treatment effects while maintaining high tumour control rates.^6^ Brachytherapy is also associated with a far shorter time commitment and fewer visits required on the part of the patient.^4^

The high dose of radiation delivered by brachytherapy can result in adverse clinical outcomes if any deviation from the prescribed radiotherapy plan occurs. Deviations can occur due to uncertainties in dose delivery following movement of OARs or radioactive source positioning and displacement, or as a result of human or equipment error. *In vivo* dosimetry has the potential to identify some of these deviations and thereby allow for their rectification.

This review aims to summarise the need for and current status of *in vivo* dosimetry for clinical brachytherapy, describe considerations for integration of *in vivo* dosimetry within routine clinical practice and propose future developments.

## The need for *in vivo* dosimetry in brachytherapy

The brachytherapy pathway involves multiple steps, each of which could potentially be associated with dosimetric uncertainty and also the potential for human or equipment error. These steps include the insertion of applicators, imaging, target delineation, applicator reconstruction, radiotherapy planning and delivery.^7^ As a high dose per fraction is delivered with HDR brachytherapy compared to EBRT, and LDR brachytherapy is usually limited to a single procedure, the potential for deviation of treatment dose from planned dose is much greater and so errors and uncertainties should be minimised where possible.^5^

The American Association of Physicists In Medicine (AAPM) classify absolute dose deviations of 10–20% and positioning differences of >5 mm between planned and actual treatments as being ‘very wrong’ and highly likely to result in a serious adverse clinical outcome.^8^ Deviations classified as ‘wrong’ include dose deviations of 5–10%, in addition to positional deviations of 3–5 mm.^8^ This includes relatively small discrepancies between the measured and delivered dose in each step in the brachytherapy treatment pathway which can cumulatively amount to clinically significant adverse events.^5^

### Dosimetric errors

#### Human and equipment error

Several aspects of the planning and delivery pathway which have the potential for human error can be mitigated by *in vivo* dosimetry. These include errors in patient setup, applicator or needle catheter placement, guide tube connections, applicator and seed reconstruction, image fusion and calculation of appropriate source time in each position, based on the residual radioactivity of the source.^5^ Administrative mistakes can also result in clinically significant errors, *e.g.* in Philadelphia when an incident occurred in 2008 in which a patient received ^125^I seeds of incorrect strength (0.38 mCi instead of 0.509 mCi) due to an error in the ordering process.^9^ This resulted in the insertion of radioactive seeds giving 25% less than the intended radiation dose.

The potential for equipment errors exists due to the complexity of brachytherapy. For example, a serious incident occurred in Indiana in 1992 when the radioactive ^192^Ir source detached from the guide wire and remained undetected in a patient receiving treatment for anal carcinoma for 5 days resulting in a substantial radiation overdose and subsequent death as a direct result of radiation exposure. More than 90 other individuals were also exposed to the radioactive source as a result of this equipment failure.^10^ Other potential errors include defects in source loading time and positions within the HDR afterloader of up to 2.0% for multiple interstitial needle applicators due to either software or motor malfunctions.^11,12^

### Dosimetric uncertainties

#### Imaging uncertainties

The quality of imaging modalities in brachytherapy can also contribute to uncertainties in brachytherapy planning. Kim et al^13^ found that random displacements of HDR prostate brachytherapy catheters by one CT slice thickness resulted in average dose errors of 0.7, 1 and 1.7% for slice thickness values of 2, 3 and 5 mm respectively. The partial volume effect, in which more than one tissue type occurs in a voxel, can result in the blurring of tissue boundaries.^14^ Uncertainties associated with pixel resolution can also impact fusion, contouring and dose reconstruction.^15^

#### Internal organ motion

For HDR gynaecological brachytherapy, there is a delay between applicator insertion, CT and/or MRI imaging, and insertion of the radioactive source during which OAR motion may occur.^16^ For LDR prostate brachytherapy, while radioactive seeds are inserted under real-time ultrasound guidance, the position of the target and OARs may change following insertion, and may have already changed in position since planning in the case of preplanning.^5^

Several studies have evaluated the movement of OARs during gynaecological brachytherapy and the resulting dosimetric impact (Table 1). Variability in findings exists, however, the majority of studies report increases in dose to the bladder and rectum as a result of OAR movement between planning and treatment delivery. Anderson et al^22^ compared planning and pre-treatment MRIs in HDR cervical brachytherapy and found >10% deviation in the minimum dose received by the most irradiated 2 cc (D2cc) of the bladder in 38.9% of fractions, rectum D2cc in 58.3% of fractions and bowel D2cc in 52.8% of fractions. Mazeron et al.,^19^ Yan et al^17^ and Rey et al^25^ found significant increases in rectal dose due to OAR movement between treatment planning and delivery, Nomden et al^21^ reported significant increases in rectal dose among outliers in their study and Lang et al^23^ found non-significant changes in rectal dose with the rectum dose constraint met in all cases.

**Table 1.** Summary of identified studies of HRCTV and OAR movement during cervical brachytherapy

Year Authors (citation)	Number of patients (fractions)	Brachytherapy type and applicator	Scans compared	Displacement (mean ± 1 SD)	Dosimetric impact (mean ± 1 SD)	Comments
2021 Yan et al.^17^	9 (38)	HDR Fletcher/Utrecht CT/MR applicator	Pre-fraction CBCT compared to planning CT	HRCTV: -2.0±3.3% Bladder: +7.9±36.7% Rectum: -6.9±34.1% Sigmoid: +19.9±68.2% Small intestine: -0.5±26.7%	HRCTV D90: -1.2±4.5% Bladder D2cc: -0.6±17.1% Rectum D2cc: +9.3±14.6% Sigmoid D2cc: +7.2±20.5% Small intestine D2cc: +1.5±12.6%	
2020 Miyasaka et al.^18^	15 (58)	HDR Fletcher CT/MRI applicator	Post-fraction CT compared to planning CT	Bladder: +65.1±84.3% Rectum: -5.9±19.0%	Bladder: D2cc: +4.6±15.1% D1cc: +3.8±15.7% D0.1cc: +4.3±20.7% Rectum : D2cc: -3.3±16.1% D1cc: -3.1±17.5% D0.1cc: -2.1±21.6%	15% dose difference: Rectum D2cc: 13.8% of total fractions Bladder D2cc: 11.1% of total fractions
2015 Mazeron et al.^19^	19 (57)	PDR Personalised vaginal mould	CTs performed prior to each fraction and compared to planning MRI	Mean intersection volume between 10 Gy isodose and OAR: Bladder: Day 1–2: -1.1±6.0 cc Day 2–3: -1.1±4.0 cc Rectum: Day 1–2: +2.0 cc Day 2–3: +0.1 cc Sigmoid: Day 1–2 +0.1±5.5 cc Day 2–3: -0.50±6.0 cc	Bladder: D2cc: +0.2±6.1%, 0.06±4.6 Gy D0.1cc: +0.5±11.9%, 0.6±12.3 Gy Rectum: D2cc: +6.3±5.6%, +3.7±3.5 Gy D0.1cc: +9.0±8.3%, +6.0±5.6 Gy Sigmoid: D2cc: +1.1±6.4%, +0.4±4.2 Gy D0.1cc: -0.4±11.6%, 1.6±10.2 Gy	Rectum D2cc increased in 17/19 patients, with 2 (10.5%) exceeding dose constraint of 75 Gy EQD2. Delivered bladder D2cc = 94 Gy (+9.1%) in one patient. Only 3/19 (15.8%) of patients had delivered D2cc within ±5% of planned dose.
2014 Simha et al.^20^	50 (50)	HDR Applicator not specified but tandem/ring visible in figures.	Pre-fraction MRI compared to pre-fraction CT	Bladder: 15.7±13.8 cc Rectum: 7.8±6.7 cc Sigmoid: 14.9±13.25 cc	Bladder D2cc: 0.5±0.4 Gy Rectum D2cc: 0.3±0.3 Gy Sigmoid D2cc: 0.6±0.6 Gy	
2014 Nomden et al.^21^	HDR: 15 (30) PDR: 10 (20)	HDR, PDR Tandem/ovoid	Pre-fraction MRI compared to post-fraction MRI compared to planning MRI	Not reported	Total estimated dose - total planned dose: HRCTV D90: -0.4±2.1 Gy Bladder D2cc: -0.3±3.8 Gy Rectum D2cc: +2.1±4.0 Gy Sigmoid D2cc: +0.9±2.9 Gy	
2013 Anderson et al.^22^	21 (36)	HDR Tandem/ring ± interstitial needles	Pre-treatment MRI compared to planning MRI	Bladder: 22.5±24.7 cc Rectum: 20.0±20.8 cc Bowel: 57.9±56.9 cc		>10% deviation from planning D2cc: Bladder: 38.9% Rectum: 58.3% Bowel: 52.8% D2cc changed by at least 10% for at least one OAR in 61% of cases Rectum D2cc: maximum absolute difference = -3.3 Gy
2013 Lang et al.^23^	21 (84)	HDR Tandem/ring	Pre-fraction MRI compared to planning MRI	Not reported	HRCTV D90: -1.2 Gy±2.7 Gy Bladder: D2cc: +0.7±4.7 Gy D0.1cc: +1.7±10.7 Gy Rectum; D2cc: +1.1±2.4 Gy 0.1 cc: +2.4±5.1 Gy Sigmoid: D2cc: -0.8±3.4 Gy D0.1cc: −1.5±8.2 Gy	Target dose constraint of ≥85 Gy EQD2 met in all cases. All differences within 3±10%
2013 Nesvacil et al.^24^	120 (363)	HDR (four centres) PDR (two centres), Tandem/ring (five centres) Tandem/ovoid (one centre) Interstitial needles (four centres)	MRI/CT compared: intrafraction (three centres) & interfraction (three centres)	Not reported	Intra fraction movement *vs* reference image: HRCTV D90: -2.5±10.8% Bladder D2cc: +1.3±17.7% Rectum D2cc: +3.8±20.5% Sigmoid D2cc: -2.3±23.5% Inter fraction movement *vs* reference image: HRCTV D90: +0.4±15.1% Bladder D2cc: -0.1±21.2% Rectum D2cc: +4.3±22.8% Sigmoid D2cc: +6.8±30.2%	
2013 Rey et al.^25^	10 (50)	HDR Interstitial needles	four models: D1 plan applied to D2 & D3 CT using updated catheter positions. Replanning performed for D2 & D3. Dwell positions/times from D2 replan applied over D3 CT & compared with D3 CT replan. Target volumes recontoured & replanned based on daily MRI.	Catheter: D1 to D2: +0.14±0.36 cm (maximum 1.11 cm) D1 to D3: +0.10±0.40 cm (maximum 1.63 cm) D2 to D3: -0.03±0.42 cm (maximum 1.85 cm)	HRCTV D90: D1 plan on D2 CT: -4.1%, SD not available. D1 plan on D3 CT: -5.7%, SD not available.	Mean D2cc rectum was significantly higher with model 1 *vs* model 3 (59.1±4.7 vs 60.9 ±4.8 Gy EQD2; *p* = 0.04). No significant difference in bladder / sigmoid D2cc.

CBCT, cone-beam computed tomography; D1/2/3, day 1,2,3; D2cc, the minimum dose received by the most irradiated 2 cc of the volume; D90, dose delivered to a minimum of 90% of the volume; HDR, high dose rate; HRCTV, high risk clinical target volume; OAR, organ at risk; PDR, pulsed dose rate

Values in Gy reported as EQD2.

In a study of 31 patients treated with pulsed dose rate (PDR) prostate brachytherapy, Dinkla et al^26^ found the distance between the prostate and the rectum as measured on CT decreased from an average of 7.1 to 5.9 mm after 24 h and to 5.3 mm after 48 h. This resulted in an increase in the rectum D2cc from planned dose of an average of 14.8% after 24 h, and 17.3% after 48 h. Similarly, due to OAR movement, the bladder D2cc increased by an average of 25.4% after 24 h and 24.8% after 48 h and the urethra D0.1cc decreased by an average of 2% after 24 h and 3.2% after 48 h. Milickovic et al^27^ evaluated urethral and rectal movement in HDR brachytherapy. The greatest movement occurred between the planning ultrasound and post-treatment ultrasound with mean movements of 1.1±1.3 mm for the urethral base and 0.4±0.4 mm for the rectum.

#### Radioactive source displacement

Studies of observed radioactive source displacement in a clinical context in gynaecological and prostate brachytherapy are summarised in Tables 1 and 2 respectively. The dosimetric impact resulting from positional displacement of radioactive sources (*e.g.* ^192^Ir) can be quantified in respect of deviations in the D90 of the high risk clinical target volume (HRCTV) in gynaecological brachytherapy. Variable impact is reported in studies, with minimal dosimetric impact in the study by Nomden et al,^21^ an intrafraction mean decrease of 2.5±10.8% in the study by Nesvacil et al,^24^ and a statistically significant mean decrease of 4.1% on the second day of brachytherapy and 5.7% on the third day compared with the original plan in the study by Rey et al.^25^

**Table 2.** Summary of identified studies of observed radioactive source and OAR movement during prostate brachytherapy

Years Authors (citation)	Number of patients (fractions)	Brachytherapy type	Scans compared	Displacement (mean ± 1 SD)	Dosimetric impact (mean ± 1 SD)	Comments
2018 Maenhout et al.^28^	17 (17)	HDR	Post-treatment MRI compared to planning MRI			Needle catheter displacement: Mean (range): X direction: 0.6 (0–2.9) mm Y direction: 0.5 (0–2.1) mm Z direction: 0.9 (0–5.5) mm Displacement >4 mm in 3 patients Median dosimetric impact: CTV D95: -0.5 Gy Urethra D10%: +0.7 Gy Rectum D1cc: -0.2 Gy Bladder D1cc: +0.1 Gy CTV D95: decreased by >2 Gy in 4 patients, up to 5.8 Gy.
2018 Buus et al.^29^	24 (48)	HDR	Pre-treatment MRI, post-treatment MRI, each compared to planning MRI	Needle catheters: 2.2±1.8 mm (pre-treatment MRI) 5.0±3.0 mm (post-treatment MRI)		Impact of displacement of needle catheters > 3 mm: (Prostate +3 mm) D90: -4.5% / mm Urethra D0.1cc: +4.0% Rectum D2cc: +8.9%
2017 Zelefsky et al.^30^	26 (26)	HDR	CBCT post-seed insertion compared to planning ultrasound	Not reported		Median prostate V100 93% (74–98%) (unadjusted). Prostate V100 <90% in 6/26 (23%) of cases (unadjusted).
2014 Kawakami et al.^31^	30 (150)	HDR	CT prior to each fraction compared to planning CT	Needle catheters: Fr 1: 6±4 mm Fr 2: 12±6 mm Fr 3: 12±6 mm Fr 4: 12±6 mm Fr 5: 12±6 mm	Not reported	
2013 Huang et al.^32^	13 (44)	HDR	Pre-treatment CT compared to planning CT	Needle catheters: 5.8±1.9 mm		Prostate D90: decreased by >10% in 8 patients, maximum: -32% (uncorrected).
2013 Dinkla et al.^26^	31	PDR	Post-treatment CT D1 (CT1), post-treatment CT D2 (CT2), post-treatment CT D3 (CT3), each compared to planning CT	Prostate: +0.0±3.9% (CT2) +0.2±4.4% (CT3) Prostate-rectum distance: -1.2 mm (CT2), SD not available -1.8 mm (CT3), SD not available	Prostate V100: -1.5±3.0% (CT2); -2.3±3.5% (CT3) Prostate D90: -3.2±4.7% (CT2); -4.2±5.3% (CT3) Rectum D2cc: +13.3±19.5% (CT2);+17.3±18.2% (CT3), Bladder D2cc: +25.4±28.1% (CT2);+24.8±30.4% (CT3) Urethra D0.1cc: -2.0±4.2% (CT2); -3.2±4.4% (CT3)	
2012 Takenaka et al.^33^	30 (210)	HDR	Post-Fr 2 CT (@21 h), post-Fr 4 CT (@45 h), post-Fr 6 CT (@69 h), each compared to planning CT	Needle catheters: Fr 2: 4.3±3.4 mm Fr 4: 4.6±4.1 mm Fr 6: 5.8±4.5 mm	CTV D100: Fr 2: 1.0±0.1% Fr 4: 1.0±0.1% Fr 6: 0.9±0.1%	CTV D90: 1.0% for all CT datasets.
2011 Foster et al.^34^	15 (30)	HDR	CBCT pre-Fr 2 compared to planning CT	Needle catheters: 5.1 mm, SD not available		Prostate V100 decreased from 93.8 to 76.2% (unadjusted). Rectal V75 increased from 0.8 to 1.5 cm^3^. No significant change in dose to bladder / urethra.
2011 Milickovic et al.^27^	25 (75)	HDR	Planning ultrasound compared to pre-treatment ultrasound compared to post-treatment ultrasound	Needle catheters: 1 mm, SD not available Urethra: Base: 0.6±0.7 mm (planning *vs* pre-treatment) 1.1±1.3 mm (planning *vs* post-treatment) Reference: 0.6±0.7 mm (planning vs pre-treatment) 0.8±0.9 mm (planning vs post-treatment) Apex: 0.6±0.8 mm (planning *vs* pre-treatment) 0.8±0.9 mm (planning *vs* post-treatment) Rectum: 0.3±0.4 mm (planning *vs* pre-treatment) 0.4±0.4 mm (planning *vs* post-treatment)	Prostate D90: -0.2 Gy (planning *vs* pre-treatment), SD not available -0.2 Gy (planning *vs* post-treatment), SD not available	Urethra: D10 exceeded 115% limit in 2 cases (post-treatment) & one case (both pre-treatment & post-treatment) Rectum: D10 exceeded 75% limit in one case (pre-treatment)
2011 Whitaker et al.^35^	25 (48)	HDR	Pre-treatment X-ray compared to planning CT		Not reported	Median catheter displacement 7.5 mm (-2.9 to +23.9 mm). 67% of implants had displacements of ≥5 mm. Displacements mostly caudal direction.
2011 Holly et al.^36^	20 (20)	HDR	Pre-treatment CBCT compared to planning CT	Needle catheters: 11 mm ±7.6 mm	1 cm displacement: Prostate V100: -20%, SD not available Prostate D90: -36%, SD not available	Prostate V100 decreased from 97.6 to 77.3% (unadjusted). Prostate D90 decreased from 110.5 to 72.9% (unadjusted). Urethra D10% increased from 118 to 125% (unadjusted).
2010 Tiong et al.^37^	91 (273)	HDR	Pre-treatment X-ray compared to planning CT	Needle catheters: 5.4±3.3 mm		82.3% of fractions had displacement >3 mm (unadjusted).
2009 Simnor et al.^38^	20 (40)	HDR	CTs prior to each fraction compared to planning CT.		Without correction: PTV D90: -27.7±22.8% (Fr 2), –32.±24.6% (Fr 3) Rectum D2cc: +0.7±0.9% (Fr 2), +0.8±1.1% (Fr 3) Urethra D30: +0.1±0.7% (Fr 2), +0.3±0.9% (Fr 3)	Needle catheter displacements relative to prostate base: Mean (range): 7.9 mm (0–21 mm) (Fr 2) 3.9 mm, (0–25.5 mm) (Fr 3) All catheters moved in a caudal direction. >70% of catheters had moved >5 mm at Fr 2. >35% of catheters had moved >5 mm at Fr 3. >20% of catheters had moved ≥12 mm.
2007 Kim et al.^39^	10 (20)	HDR	CT pre-FR 2 compared to planning CT		Not reported	Caudal displacement of needle catheters: Mean (range): 5.4 mm (-3.8 to 18.0 mm) (reference: prostatic markers) 2.7 mm (-6.0 to 13.5 mm) (reference: bony landmark)
2006 Pieters et al.^40^	31	PDR	Post-treatment CT D2, post- treatment CT D3, each compared to planning CT			Needle catheter displacement: Mean (range) 1.0 mm (0–6 mm) D2 1.2 mm (0–6 mm) D3 Mean dosimetric impact D1 compared to D3: mean (95% CI): Prostate V100: -0.3 ml (0.1–0.5) Urethra D0.5cc: 1.0 cGy/pulse (0.0–2.0) Rectum D2cc: 0.9 cGy/pulse (0.3–1.6)
2004 Mullokandov et al.^41^	50 (100)	HDR	Pre-treatment CT at varying intervals compared to planning CT			Caudal displacement of needle catheters: Mean (range) 2 mm (0–4 mm) (pre-Fr 2) 8 mm (5–14 mm) (pre-Fr 3) 10 mm (5–23 mm) (pre-Fr 4) Mean overall displacement: 9 mm Median dosimetric change between Fr 1 and Fr 3: Prostate D90: -35% (0 to – 60%) Minimal dose to prostate base: -35% (-17 to -65%) D1cc prostate: -13% (- three to -19%)
2003 Hoskin et al.^42^	20 (40)	HDR	CT pre-Fr 2 compared to planning CT		Maximum urethral dose: +1.1 Gy, SD not available (unadjusted) Maximum rectal dose: +0.2 Gy, SD not available (unadjusted)	Needle catheter displacement: Mean (range): Caudal: 11.5 mm (0–42 mm) Range of dosimetric impact to prostate: D90: 99.5 to 63.6% (unadjusted) V100: 89.3 to 69.5% (unadjusted)
2002 Beaulieu et al.^43^	35 (35)	LDR	Pre-treatment ultrasound compared to planning ultrasound			Only 13/35 cases had relatively constant volumes with <5% variation (significant changes up to 30%). Dosimetric impact on prostate V100: -5.7% (up to -20.9%)
2001 Martinez et al.^44^	10 (40)	HDR	Ultrasound prior to any needle manipulation, pre-Fr 1, post-Fr 4	Needle catheter adjustment: 20 mm, SD not available (Fr 2 *vs* Fr 1) 4 mm, SD not available (Fr 3 *vs* Fr 2) 4 mm, SD not available (Fr 4 *vs* Fr 3)		Dosimetric impact: Mean (range): Prostate D90: -4% (-19 to +8%) (Fr 4 compared to Fr 1) Urethral D10: +10% (0 to +18%) (Fr 4 compared to Fr 1)
2000 Damore et al.^45^	96 (384)	HDR	Pre-treatment X-ray compared to planning X-ray		Not reported	All caudal needle catheter displacements. Needle catheter displacements: Mean (maximum): Implant needles: 7.6 mm (28.5 mm) Gold marker seeds: 3.6 mm (11.4 mm) At least 1 cm caudal displacement in 15.5% of cases.

CBCT, cone-beam computed tomography; CI, confidence interval; CTV, clinical target volume; D10, dose delivered to a minimum of 10% of the volume; D30, dose delivered to a minimum of 30% of the volume; D90, dose delivered to a minimum of 90% of the volume; D95, dose delivered to a minimum of 95% of the volume; D1/2/3, day 1/2/3; D1cc, the minimum dose received by the most irradiated 1cc of the volume; D2cc, the minimum dose received by the most irradiated 2cc of the volume; D0.5cc, the minimum dose received by the most irradiated 0.5cc of the volume; Fr, fraction; HDR, high dose rate; LDR, low dose rate; OAR, organs at risk; PDR, pulsed dose rate; US, ultrasound; V75, volume receiving 75% of prescription dose; V100, volume receiving 100% of prescription dose.

The majority of catheter displacements during prostate brachytherapy tend to occur in a caudal direction relative to the prostate gland. In a study of 20 patients receiving HDR prostate brachytherapy, Simnor et al^38^ found more than 70% of catheter needles had moved >5 mm in the caudal direction by the second fraction and more than 35% had moved >5 mm by the third fraction, with more than 20% of catheters in total moving ≥12 mm. Without correction, these displacements would have resulted in a mean 28% decrease in the D90 to the planning target volume (PTV) in the second fraction, and a mean 32% decrease in D90 PTV in the third fraction. Holly et al^36^ found an average displacement of 11 mm to result in >20% decrease in V100 and 38% decrease in prostate D90 with a corresponding increase in the mean minimum dose delivered to the most irradiated 10% of the urethra (D10%) from 118 to 125%. In a study of pre-planned LDR prostate brachytherapy, Beaulieu et al^43^ found only 13 of 35 studied cases had relatively constant volumes with <5% variation with significant changes up to 30% and a resulting mean dosimetric impact on prostate V100 of -5.7%, up to -20.9%.

Several studies have systematically manually displaced the position of radioactive sources in brachytherapy plans to determine the threshold of movement for significant dosimetric impact and are summarised in Table 3. Hoskin et al^49^ report a 5% decrease in prostate D90 and V100 to be associated with a 10% increase in biochemical failure in prostate brachytherapy. Poder et al^46^ found the proportion of plans which demonstrated at least a 5% reduction in target coverage parameters increased with displacements >2 mm. The study found that the target minimum V100 was not met in 75% of plans following a 3 mm shift in the cranial direction, in 50% of plans following a 3 mm shift in the caudal direction, and in 35% of plans following a 3 mm shift in the medial direction. In a study of HDR cervical brachytherapy plans, Tanderup et al^48^ found the HRCTV dose–volume histogram (DVH) shifted by a mean of approximately 2% per mm shift in the lateral and longitudinal directions, and by approximately 1.5% per mm shift in the anterior and posterior directions. The D2cc of the bladder and rectum changed by approximately 5% per mm shift in the anterior and posterior directions and the D0.1cc of the same OARs changed by approximately 6% per mm shift in the same directions.

**Table 3.** Summary of identified studies of manual source displacement in pelvic brachytherapy

Year Authors (citation)	Number of patients	Brachytherapy type	Location of displacements	Magnitude of displacements	Pertinent dosimetric results
2019 Poder et al.^46^	20	HDR prostate	three catheters displaced: 3 most heavily weighted 3 closest to urethra & rectum in direction of OAR	CC: ±1–6 mm Transverse: ±1–6 mm AP: ±1–6 mm	Positioning errors most sensitive in CC direction. Positioning errors more sensitive in cranial *vs* caudal & lateral *vs* medial directions. 5% change in prostate D90 & V100 with errors of ≈ 3 mm. % failing prostate V100 goal by >5% increases when error >2 mm. % plans failing prostate V100 goal with 3 mm shift per direction: Cranial: 75%; Caudal: 50% Posterior: 0%; Anterior: 5% Medial: 35%; Lateral: 10% Urethra D1cc: >50% fail with 2 mm error in medial, anterior & posterior directions. Rectum D2cc:>50% fail with 5 mm error in posterior direction.
2011 Kolkman-Deurloo et al.^47^	5	HDR prostate	All catheters displaced as a single unit Central, most ventral or most dorsal catheter rows displaced	Caudal: 3, 5, 7, 10 mm Caudal: 5 mm	Prostate V100: 91.4% (3 mm), 87.2% (5 mm), 82.6% (7 mm), 75.3% (10 mm). Rectum V80 exceeded tolerance in 80% of cases for all displacements. Urethra V120 increased by a factor ranging from negligible to 26.
2010 Tiong et al.^37^	20	HDR prostate	All catheters displaced as a single unit	Caudal: 3, 6, 9, 12 mm	Median TCP: 0.998 (3 mm), 0.964 (6 mm), 0.797 (9 mm), 0.265 (12 mm). Only 75% of 6 mm displacement plans had TCP >95%.
2008 Tanderup et al.^48^	20: 10 ring & tandem intracavitary 10 interstitial & intracavitary	HDR cervix	Entire applicator displaced	Intracavitary: CC: ±3 mm,±5 mm Transverse:±3 mm AP: ±3 mm Rotation:±15° (4 mm) Interstitial & intracavitary: CC: ±3 mm,±5 mm	Intracavitary: HRCTV D90: mean change of ≈ 2% / mm for lateral & CC directions, ≈ 1.5% / mm in AP direction. Bladder & rectum D2cc: mean change of 5% / mm in AP direction. Bladder & rectum D0.1cc: mean change of 6% / mm in AP direction. Rotation had limited impact. Interstitial & intracavitary: Sigmoid D0.1cc CC displacement 2.9% / mm ( *vs* 1.9% / mm intracavitary).

AP, anteroposterior; CC, craniocaudal; D1cc, the minimum dose received by the most irradiated 1 cc of the volume; D0.1cc, the minimum dose received by the most irradiated 0.1 cc of the volume; D2cc, the minimum dose received by the most irradiated 2 cc of the volume; D90, dose delivered to a minimum of 90% of the volume; HDR, high dose rate; HRCTV, high risk clinical target volume; TCP, tumour control probability; V80, volume receiving 80% of prescription dose; V100, volume receiving 100% of prescription dose; V120, volume receiving 120% of prescription dose

It is clearly important that the potential movement of all radioactive sources and OARs during brachytherapy is considered given the potential clinical impact geometric and dosimetric uncertainties can have.^5^ Displacements as small as 3 mm have been shown to have significant outcomes on brachytherapy dosimetry and target coverage in studies.

## Current status of *in vivo* dosimetry in brachytherapy

*In vivo* dosimetry consists of real-time monitoring of radioactive source placement and dose during the delivery of radiotherapy. This involves the placement of radiation detectors in the vicinity of radioactive sources within the body, which relay the measured dose to the clinical staff and hence allows for comparison of calculated radiotherapy dose with actual dose delivered.^11^ *In vivo* dosemeters, therefore, allow for independent verification of brachytherapy delivery, comparison of institutional practice and quality assurance of radiotherapy treatment provision resulting in safer, more accurate clinical practice.^50^ This is of particular importance with the delivery of high brachytherapy doses, *e.g.* the delivery of a boost to the dominant intraprostatic lesion seen on MRI, which has been explored in recent studies.^51,52^ With increasing dose, the potential for adverse effects also increases and so precise accurate dose assessment is vital.^52^

The inclusion of *in vivo* dosimetry in clinical practice has been hesitant, due to a lack of affordable, efficient, commercially available dosemeters. The requirements for precision, stability and dosemeter positioning certainty are additional challenges that limit the routine adoption of *in vivo* dosimetry in clinical practice.^53^ Most studies to date focus on pre-clinical models demonstrating proof of concept,^54–56^ although some clinical studies have been performed in pelvic brachytherapy. Dosemeters in the form of metal-oxide-semiconductor field-effect transistors (MOSFET), optical fibres and semiconductors have been tested clinically, all within HDR brachytherapy settings, with dosemeters inserted in the rectum, urinary catheter or within the brachytherapy target. While several are commercially available,^57–59^ they are not routinely used in brachytherapy clinical practice.^11,53^ Limitations include angular and energy dependence of semiconductor diodes, energy dependence and limited lifespan of MOSFETs and Cerenkov light production in optical fibre dosemeters.^53^

Belley et al^60^ evaluated the feasibility and effectiveness of a nanoscintillator-based fibre-optic dosemeter (nanoFOD) compared to thermoluminescent dosemeters (TLD) in vaginal cylinder HDR brachytherapy. The dosemeter was adhered to the cylinder at a fixed distance, to which two TLDs were also attached to provide reference measurements. Real-time data were available for 27 fractions among 9 participants. The fibre-optic dosemeter readings were comparable to TLD measurements and 63% of measurements with the fibre-optic dosemeter were within 5% of the treatment planning system (TPS) (compared with 70% of TLD measurements), 26% were within 5–10% (22% of TLD measurements) and 11% were within 10–20% (7% of TLD measurements), with a median ratio of nanoFOD/TPS dose of 1.00 (IQR 0.94–1.02). The use of TLD as a reference standard demonstrated feasibility of the nanoFOD within a clinical setting.

In a study of a radioluminescent crystal dosemeter placed within a dedicated brachytherapy catheter during HDR brachytherapy Johansen et al^61^ found measured compared with planned doses to differ by a mean of -4.7% (range -17 to +12%) with mean shifts of brachytherapy needles of 0.2±1.1 mm (radial) and 0.3±2.0 mm (longitudinal). Limitations of the study included the measurement of displacements relative to the radioluminescent crystal rather than to patient anatomy and the use of only one dosemeter. Integration of *in vivo* dosemeters with imaging systems and the use of an array of dosemeters would reduce positional uncertainty.^62^ Additional studies of the clinical use of *in vivo* dosimetry in pelvic brachytherapy are summarised in Table 4.

**Table 4.** Summary of identified clinical studies using *in vivo* dosimetry in pelvic brachytherapy

Year Authors (citation)	Type of dosemeter	Clinical application	Location of detector	Differences between calculated and measured doses
2021 Hayashi et al.^58^	Optically stimulated luminescence	HDR cervix	Rectal probe	Mean +3.9 (±12.7% SD)
2020 Mason et al.^63^	MOSFET	HDR prostate	Brachytherapy needle in prostate	Mean +5.2% (range -17.3% to +7.4%)
2020 Poder et al.^64^	MOSkin (MOSFET)	HDR prostate	Rectal probe	Mean 0.3% (±11.6% SD)
2020 Jamalludin et al.^59^	MOSkin (MOSFET) and PTW 9112 semiconductor	HDR cervix	Rectal probe	MOSkin: Mean −3.2% (±10.1% SD); PTW 9112: Mean −15.5% (±9.7% SD)
2018 Johansen et al.^61^	Optical fibre	HDR prostate	Brachytherapy needle in prostate	Mean -4.7% (range -17 to +12%)
2018 Belley et al.^60^	Optical fibre / thermoluminescence	HDR vagina	Lateral surface of vaginal cylinder	63% of measurements were within 5% of TPS; 26% within 5–10%; 11% within 10–20%
2017 Carrara et al.^65^	MOSkin (MOSFET)	HDR vagina	Rectal probe	Mean +2.2% (±6.9% SD)
2017 Wagner et al.^66^	Alanine/electron spin resonance	HDR prostate	Urinary catheter	Mean -2.4 Gy (range -7.9 to +0.2 Gy)
2017 Van Gellekom et al.^67^	MOSFET	HDR vagina	Vaginal applicator needle	Mean +3% (±14% SD)
2016 Carrara et al.^68^	MOSkin (MOSFET)	HDR prostate	Rectal probe	Mean +6.7% (range ±5.1% SD)
2016 Mason et al.^69^	MOSFET	HDR prostate	Brachytherapy needle in prostate	Mean -6.4% (range +5.1 to 15.2%)
2014 Zaman et al.^70^	Semiconductor diode	HDR cervix	Rectal probe	Range -8.5% to +41.2%
2013 Sharma et al.^(93)^	Optically stimulated luminescence	HDR cervix	Rectal retractor	Range -14.9% to +13.7%
2012 Allahverdi et al.^71^	Semiconductor diode	HDR cervix	Rectal probe	Mean 6.5% (range -22 to +39%)
2011 Suchowerska et al.^(94)^	Optical fibre	HDR prostate	Urinary catheter	≤9%

HDR, high dose rate; MOSFET, metal-oxide-semiconductor field-effect transistor

While the magnitude of what constitutes a clinically acceptable deviation is variable and specific to each patient site, it is essential for clinically useful *in vivo* dosemeters to detect deviations classified as ‘wrong’ by the AAPM (dose distribution and delivery deviations of 5% and positioning deviations of 3 mm) and the ideal is for detection sensitivity to be as high as possible.^8^ Currently, the accuracy of *in vivo* dosimetry systems varies significantly with mean differences between calculated and measured radiation dose for MOSFET, optically stimulated dosemeters and semiconductors of up to 6.7, 4.7 and 15.5% respectively (Table 4).

## Clinical considerations

Several clinical considerations are necessary in order to overcome the current limitations associated with the integration of *in vivo* dosimetry into routine clinical brachytherapy.

### Workflow

Service and resource pressures as well as the existing complexities of brachytherapy procedures are potential barriers to the practical implementation of *in vivo* dosimetry.^72^ In addition, the greater the time between imaging and treatment delivery, the greater the risk of internal organ motion and increased positional uncertainties.^65^ Integration with the existing patient workflow, *e.g.* affixing the *in vivo* dosemeters to the afterloading device in HDR gynaecological brachytherapy, is preferable, to avoid the need for additional procedures.^53^

### Dosemeter placement

Important considerations during *in vivo* dosimetry are the accuracy, reproducibility and stability of dosemeter placement. Appropriate fixation must take place to ensure no movement occurs between dosemeter insertion and delivery of radiotherapy. Waldhäusl et al report dosemeter probe shifts as small as 2.5 mm result in measured dose differences of >10%.^73^ Due to the steep dose fall off associated with brachytherapy, dosemeter movement of only a few millimetres can result in erroneous dose measurements, the triggering of false alarms and the failure to detect radiotherapy dose deviations. This requirement for accurate placement of *in vivo* dosemeters can limit their practical use.^4^ Therefore, dosemeters must be used in conjunction with imaging techniques to ensure adequate localisation. In addition, the insertion process of *in vivo* dosemeters should be integrated with existing equipment such as urinary catheters and applicators to minimise risks of bleeding and infection.^53^

#### Sensitivity and specificity

The sensitivity and specificity of dosemeters are other important considerations. In the context of *in vivo* dosimetry, sensitivity is the likelihood that a dosemeter will detect errors in dose or positioning if these errors exist.^71^ Use of a dosemeter with high sensitivity, therefore, should detect any dosimetric errors that occur and confirm the absence of such errors if no alarm sounds. Specificity is the likelihood that when a dosemeter signals an error in dose or positioning, that this is a true error and not a ‘false alarm’.^71^ A balance must be struck to ensure that the vast majority of errors are detected without the expense of triggering excessive false alarms. Dosemeter susceptibility to external factors and environmental influences including humidity, temperature, direction, angular dependence and energy dependence are important to consider and these influences should be minimised where possible, or at least correction factors clearly documented.^50^ The atomic number of the chosen dosemeter should be similar to water to reduce energy dependence.^50^

#### Cost

Significant costs are associated with the implementation and use of *in vivo* dosimetry in radiotherapy.^74^ Many hospitals and healthcare systems have limited budgets and it is imperative that the dosemeters are cost-effective.^75,76^

## Future of *in vivo* dosimetry

Time-resolved, or real-time dosimetry, has the potential to significantly reduce brachytherapy errors. Triggering an alarm during the delivery of radiation in brachytherapy signifies to the clinical team that an error has occurred and prompts immediate investigation and resolution of this error. This may result in treatment interruptions, prolonging of treatment times and may cause discomfort for the patient in addition to increasing the complexity of the procedure for clinical staff who must compensate for the detected dose error.^77^ Integration of *in vivo* dosemeters with treatment planning software to allow for real-time monitoring of radiation dose delivery and distribution could allow for the brachytherapy plan to be adapted in such a manner as to compensate for any significant dose deviations. Such advances are dependent on high precision and accuracy of *in vivo* dosimetry and advanced software development but would minimise any additional treatment time and clinical staff workload as a result of errors in radiation dose and distribution.

In the future, *in vivo* dosemeters could also facilitate uptake in radiobiology-guided brachytherapy. Hypoxia is associated with radiotherapy resistance and inferior clinical outcomes.^78^ It is commonly associated with solid tumours due to their immature, disorganised vascular supply which develops as a result of overexpression of pro-angiogenic factors.^79^ Movsas et al^80^ measured pO2 in human prostate carcinomas using Eppendorf microelectrodes and found these to be significantly lower than the pO2 present in normal muscle controls, with increasing hypoxia associated with increasing clinical stage. The ratio of prostate to normal muscle pO2 was the strongest predictor for biochemical control. Similar results were found in studies by Turaka et al^81^ of prostate cancer, and by Rofstad et al^82^ of cervical cancer, demonstrating the need for adaptive strategies to target hypoxia within tumours.

Dose escalation within identified areas of tumour hypoxia is a potential method by which the negative effect of hypoxia on tumour control can be overcome.^83^ Hypoxic sensors have been described in the literature including the aforementioned Eppendorf oxygen electrode,^80^ a fibre-optic sensor using ruthenium luminophore incorporated into a silicone rubber polymer tip,^84^ fluorescent peptide probes based on the oxygen-dependent degradation domain of HIF-1α,^85^ imaging such as blood-oxygen-level dependent (BOLD) functional MRI which evaluates changes in signal intensity between diamagnetic oxyhaemoglobin and paramagnetic deoxyhaemoglobin,^86^ and PET/CT using hypoxia-specific tracers such as ^18^F-fluoromisonidazole (^18^F-FMISO).^87^ Integration of a hypoxic sensor within an *in vivo* dosemeter would allow for tumour hypoxia to be mapped and measured in real-time with dose escalation to these areas.

A similar approach could be taken with dosemeters which detect the presence of DNA double-strand breaks (DSBs). DSBs are a critical from of DNA damage and, if not correctly repaired, are an important mechanism by which radiation induces cell death.^88^ A pre-clinical model consisting of magnetic streptavidin beads attached to four kilobase pair DNA strands has shown promising results in the detection of DNA DSBs.^89^ Detecting these DSBs in real-time during brachytherapy would provide the opportunity to adapt the dose depending on their quantity and location. For example, increased dose could be delivered in areas with minimal DSBs with reduced dose in areas with many DSBs.

Brachytherapy plans adapted to tumour hypoxia and DSBs would enable dose escalation in areas of radioresistance and reduction in areas of radiosensitivity. By detecting these, *in vivo* dosemeters have the potential to provide for a personalised radiotherapy approach in real-time adaptive brachytherapy which should result in improved outcomes in terms of tumour control and toxicities for patients.

## Conclusion

Prostate and gynaecological brachytherapy have increased in complexity in recent years, due to advances in imaging, techniques and software. Adaptive brachytherapy has the potential to optimise target dose distribution, reduce side-effects from treatment and introduces the potential for dose escalation. It is important that the radiation doses delivered to the brachytherapy target and OARs are accurately measured and documented, and *in vivo* dosimetry provides an opportunity for adaptive brachytherapy in real-time. There are several important considerations regarding the practicalities of *in vivo* dosimetry, which must be addressed prior to its incorporation into routine clinical practice, and the benefits of the procedure must outweigh any potential risks to the patient. The EU Horizon 2020 Origin project^90^ is working to address the current barriers to the clinical implementation of *in vivo* dosimetry and to develop a real-time system based on optical fibre-based sensing technology for use in prostate and gynaecological brachytherapy.
